# (4-Hydr­oxy-2-oxidobenzaldehyde thio­semicarbazonato-κ^3^ *O* ^2^,*N* ^1^,*S*)(1,10-phenanthroline-κ^2^ *N*,*N*′)zinc(II) dimethyl sulfoxide disolvate monohydrate

**DOI:** 10.1107/S160053680804169X

**Published:** 2008-12-13

**Authors:** Kong Wai Tan, Chew Hee Ng, Mohd Jamil Maah, Seik Weng Ng

**Affiliations:** a Department of Chemistry, University of Malaya, 50603 Kuala Lumpur, Malaysia; b Faculty of Engineering and Science, Universiti Tunku Abdul Rahman, 53300 Kuala Lumpur, Malaysia; c Department of Chemistry, University of Malaya, 50603 Kuala Lumpur, Malaysia

## Abstract

The Zn^II^ atom in the title compound, [Zn(C_8_H_7_N_3_O_2_S)(C_12_H_8_N_2_)]·2C_2_H_6_OS·H_2_O, is *N*,*N*′-chelated by the *N*-heterocycle and *N*,*O*,*S*-chelated by the deprotonated Schiff base in a distorted square-pyramidal enviroment. Hydrogen bonds link the mononuclear mol­ecule, the water and the dimethyl sulfoxide (DMSO) mol­ecules into a linear chain motif. One DMSO mol­ecule is disordered over two positions in respect of the S atom in an approximate 1:1 ratio.

## Related literature

For reports of the metal derivatives of 2,4-dihydroxy­benzaldehyde thio­semicarbazone, see: Broomhead & Dwyer (1961 ▶); Gingras *et al.* (1960 ▶); Liu *et al.* (1974 ▶); Luo *et al.* (1988 ▶); Mayadeo *et al.* (1986 ▶); Onuska *et al.* (1996 ▶); Shen & Li (2006 ▶); Zhu *et al.* (1991*a* ▶,*b* ▶).
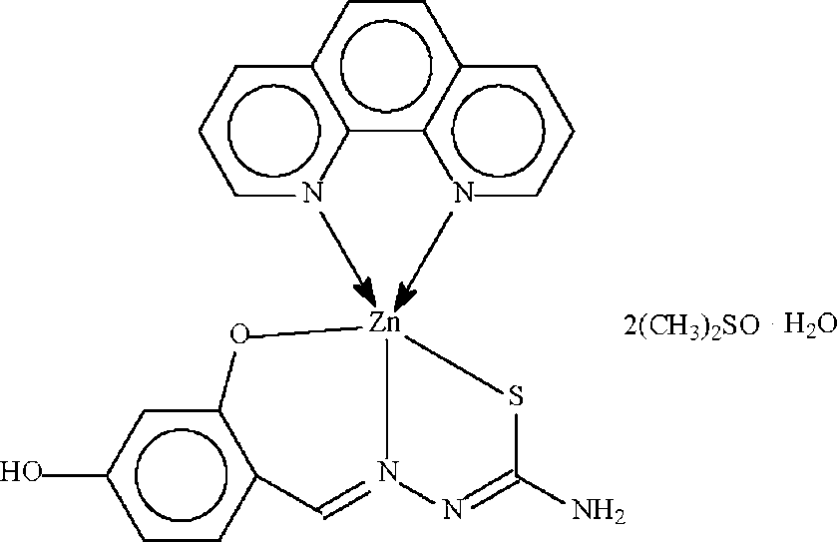


## Experimental

### 

#### Crystal data

[Zn(C_8_H_7_N_3_O_2_S)(C_12_H_8_N_2_)]·2C_2_H_6_OS·H_2_O
*M* *_r_* = 629.07
Triclinic, 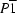

*a* = 9.3582 (5) Å
*b* = 9.8181 (5) Å
*c* = 15.2913 (8) Å
α = 73.641 (3)°
β = 82.482 (4)°
γ = 88.059 (4)°
*V* = 1336.5 (1) Å^3^
*Z* = 2
Mo *K*α radiation
μ = 1.20 mm^−1^
*T* = 100 (2) K
0.05 × 0.01 × 0.01 mm

#### Data collection

Bruker SMART APEX diffractometer
Absorption correction: multi-scan (*SADABS*; Sheldrick, 1996 ▶) *T*_min_ = 0.943, *T*_max_ = 0.988
12760 measured reflections
6125 independent reflections
3397 reflections with *I* > 2σ(*I*)
*R*_int_ = 0.097

#### Refinement

*R*[*F* ^2^ > 2σ(*F* ^2^)] = 0.074
*wR*(*F* ^2^) = 0.211
*S* = 0.98
6125 reflections
344 parameters
27 restraints
H-atom parameters constrained
Δρ_max_ = 1.51 e Å^−3^
Δρ_min_ = −1.29 e Å^−3^

### 

Data collection: *APEX2* (Bruker, 2007 ▶); cell refinement: *SAINT* (Bruker, 2007 ▶); data reduction: *SAINT*; program(s) used to solve structure: *SHELXS97* (Sheldrick, 2008 ▶); program(s) used to refine structure: *SHELXL97* (Sheldrick, 2008 ▶); molecular graphics: *X-SEED* (Barbour, 2001 ▶); software used to prepare material for publication: *publCIF* (Westrip, 2009 ▶).

## Supplementary Material

Crystal structure: contains datablocks I, global. DOI: 10.1107/S160053680804169X/sj2562sup1.cif

Structure factors: contains datablocks I. DOI: 10.1107/S160053680804169X/sj2562Isup2.hkl

Additional supplementary materials:  crystallographic information; 3D view; checkCIF report

## Figures and Tables

**Table 1:** Hydrogen-bond geometry (Å, °)

*D*—H⋯*A*	*D*—H	H⋯*A*	*D*⋯*A*	*D*—H⋯*A*
O2—H2⋯O3	0.84	1.84	2.679 (6)	172
N3—H31⋯N2^i^	0.88	2.11	2.984 (8)	174
N3—H32⋯O4	0.88	2.33	3.074 (8)	143
O1w—H1w1⋯O1^ii^	0.84	2.11	2.911 (7)	160
O1w—H1w2⋯O2	0.84	2.25	3.055 (7)	161

Symmetry codes: (i) 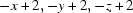; (ii) 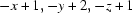.

**Fig. 1.**
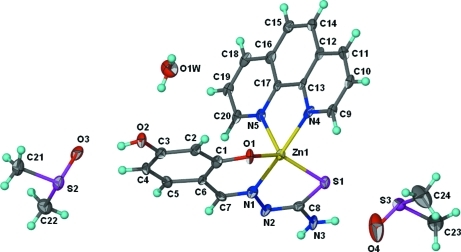
Thermal ellipsoid (Barbour, 2001) plot of Zn(C12H8N2)(C8H7N3O2S).2DMSO.H2O at the 70% probability level. Hydrogen atoms are drawn as spheres of arbitrary radii. The disorder in the DMSO is not shown.

## supplementary crystallographic information

### Experimental

Zinc acetate monohydrate (0.22 g, 1 mmol), 2,4-dihydroxybenzaldehyde thiosemicarbazone (0.21 g, 1 mmol) and 1,10-phenanthroline (0.20 g, 1 mmol) were heated in ethanol (50 ml) to give a yellow solution. The compound that separated on cooling the solution was recrystallized from DMSO.

### Refinement

Carbon-bound H-atoms were placed in calculated positions (C—H 0.95– to 0.98 Å) and were included in the refinement in the riding model approximation, with *U*(H) set to 1.2–1.5*U*_eq_(C). The amino and hydroxy H-atoms were similarly placed. The water H-atoms were placed in chemically sensible positions on the basis of hydrogen bonding but were not refined. Their temperature factors were similarly tied.

One of the two DMSO molecules is disodered in the sulfur atom only. The disorder refined to a 0.56 (1):0.44 ratio.

The middle six-membered ring of the phenanthroline unit was refined as a rigid hexagon of 1.39 Å sides. The N4 and C12 atoms of the unit were restrained to be nearly isotropic; a tight restraint was used.

The final difference Fourier map had a peak in the vicinity of N1 and a hole in the vicinity of Zn1.

### Crystal data

**Table:**

[Zn(C_8_H_7_N_3_O_2_S)(C_12_H_8_N_2_)]·2C_2_H_6_OS·H_2_O	*Z* = 2
*M**_r_* = 629.07	*F*(000) = 652
Triclinic, *P*1	*D*_x_ = 1.563 Mg m^−^^3^
Hall symbol: -P 1	Mo *K*α radiation, λ = 0.71073 Å
*a* = 9.3582 (5) Å	Cell parameters from 932 reflections
*b* = 9.8181 (5) Å	θ = 2.2–21.9°
*c* = 15.2913 (8) Å	µ = 1.20 mm^−^^1^
α = 73.641 (3)°	*T* = 100 K
β = 82.482 (4)°	Prism, yellow
γ = 88.059 (4)°	0.05 × 0.01 × 0.01 mm
*V* = 1336.5 (1) Å^3^	

### Data collection

**Table:**

Bruker SMART APEX diffractometer	6125 independent reflections
Radiation source: fine-focus sealed tube	3397 reflections with *I* > 2σ(*I*)
graphite	*R*_int_ = 0.097
ω scans	θ_max_ = 27.5°, θ_min_ = 1.4°
Absorption correction: multi-scan (*SADABS*; Sheldrick, 1996)	*h* = −12→12
*T*_min_ = 0.943, *T*_max_ = 0.988	*k* = −12→12
12760 measured reflections	*l* = −19→19

### Refinement

**Table:**

Refinement on *F*^2^	Primary atom site location: structure-invariant direct methods
Least-squares matrix: full	Secondary atom site location: difference Fourier map
*R*[*F*^2^ > 2σ(*F*^2^)] = 0.074	Hydrogen site location: inferred from neighbouring sites
*wR*(*F*^2^) = 0.211	H-atom parameters constrained
*S* = 0.98	*w* = 1/[σ^2^(*F*_o_^2^) + (0.0989*P*)^2^] where *P* = (*F*_o_^2^ + 2*F*_c_^2^)/3
6125 reflections	(Δ/σ)_max_ = 0.001
344 parameters	Δρ_max_ = 1.51 e Å^−^^3^
27 restraints	Δρ_min_ = −1.29 e Å^−^^3^

### Fractional atomic coordinates and isotropic or equivalent isotropic displacement parameters (Å^2^)

**Table:**

	*x*	*y*	*z*	*U*_iso_*/*U*_eq_	Occ. (<1)
Zn1	0.78197 (8)	1.15841 (8)	0.72088 (5)	0.0148 (2)	
S1	0.86430 (18)	1.30637 (17)	0.80123 (11)	0.0196 (4)	
S2	0.30700 (19)	0.28436 (18)	0.79460 (12)	0.0228 (4)	
S3	1.2635 (3)	1.5316 (3)	0.9107 (2)	0.0249 (10)	0.564 (6)
S3'	1.1708 (4)	1.5622 (4)	0.9722 (3)	0.0235 (13)	0.436 (6)
O1	0.6115 (5)	1.0571 (4)	0.7064 (3)	0.0183 (10)	
O2	0.4103 (5)	0.6534 (5)	0.6604 (3)	0.0206 (10)	
H2	0.4048	0.5648	0.6818	0.031*	
O3	0.4176 (5)	0.3697 (5)	0.7213 (3)	0.0254 (11)	
O4	1.1918 (7)	1.4255 (6)	0.9740 (4)	0.067 (2)	
O1W	0.5152 (5)	0.8171 (5)	0.4620 (4)	0.0352 (13)	
H1W1	0.4596	0.8455	0.4217	0.053*	
H1W2	0.4664	0.7745	0.5118	0.053*	
N1	0.8024 (6)	1.0029 (6)	0.8431 (4)	0.0180 (12)	
N2	0.8839 (6)	1.0295 (6)	0.9059 (4)	0.0173 (12)	
N3	0.9991 (6)	1.1973 (6)	0.9465 (4)	0.0198 (12)	
H31	1.0275	1.1297	0.9922	0.024*	
H32	1.0243	1.2860	0.9385	0.024*	
N4	0.7906 (6)	1.3147 (6)	0.5889 (4)	0.0169 (12)	
N5	0.9576 (5)	1.0814 (5)	0.6433 (4)	0.0141 (11)	
C1	0.6018 (7)	0.9163 (7)	0.7315 (4)	0.0166 (14)	
C2	0.5111 (6)	0.8529 (6)	0.6879 (4)	0.0147 (13)	
H2A	0.4559	0.9117	0.6439	0.018*	
C3	0.4999 (7)	0.7065 (7)	0.7073 (4)	0.0177 (14)	
C4	0.5791 (7)	0.6167 (7)	0.7718 (4)	0.0182 (14)	
H4	0.5747	0.5165	0.7834	0.022*	
C5	0.6633 (7)	0.6775 (7)	0.8177 (4)	0.0174 (14)	
H5	0.7162	0.6171	0.8624	0.021*	
C6	0.6750 (7)	0.8259 (6)	0.8015 (4)	0.0144 (13)	
C7	0.7622 (6)	0.8737 (7)	0.8587 (4)	0.0152 (13)	
H7	0.7915	0.8055	0.9108	0.018*	
C8	0.9168 (7)	1.1651 (7)	0.8889 (4)	0.0163 (14)	
C9	0.7054 (7)	1.4278 (6)	0.5623 (5)	0.0173 (14)	
H9	0.6259	1.4427	0.6040	0.021*	
C10	0.7303 (7)	1.5230 (7)	0.4761 (4)	0.0180 (14)	
H10	0.6672	1.6012	0.4589	0.022*	
C11	0.8459 (7)	1.5049 (7)	0.4152 (5)	0.0194 (14)	
H11	0.8637	1.5712	0.3563	0.023*	
C12	0.9394 (4)	1.3858 (3)	0.4409 (3)	0.0162 (13)	
C14	1.0583 (4)	1.3616 (4)	0.3825 (2)	0.0191 (14)	
H14	1.0802	1.4255	0.3230	0.023*	
C15	1.1451 (4)	1.2440 (4)	0.4113 (2)	0.0189 (14)	
H15	1.2264	1.2274	0.3714	0.023*	
C16	1.1131 (4)	1.1505 (3)	0.4984 (3)	0.0180 (14)	
C17	0.9942 (4)	1.1746 (4)	0.5568 (2)	0.0143 (13)	
C13	0.9074 (4)	1.2923 (4)	0.5280 (2)	0.0147 (13)	
C18	1.1962 (7)	1.0255 (7)	0.5292 (5)	0.0184 (14)	
H18	1.2784	1.0062	0.4911	0.022*	
C19	1.1562 (6)	0.9336 (7)	0.6142 (4)	0.0164 (14)	
H19	1.2095	0.8493	0.6353	0.020*	
C20	1.0360 (7)	0.9656 (6)	0.6694 (5)	0.0167 (14)	
H20	1.0093	0.9013	0.7283	0.020*	
C21	0.2630 (8)	0.1380 (7)	0.7548 (5)	0.0269 (16)	
H21A	0.2223	0.1737	0.6967	0.040*	
H21B	0.1922	0.0768	0.8007	0.040*	
H21C	0.3503	0.0834	0.7452	0.040*	
C22	0.4045 (8)	0.1879 (8)	0.8850 (5)	0.0302 (17)	
H22A	0.4803	0.1322	0.8607	0.045*	
H22B	0.3387	0.1240	0.9330	0.045*	
H22C	0.4483	0.2545	0.9110	0.045*	
C23	1.3333 (8)	1.6563 (9)	0.9636 (6)	0.040 (2)	
H23A	1.2790	1.6460	1.0245	0.060*	0.564 (6)
H23B	1.3231	1.7535	0.9249	0.060*	0.564 (6)
H23C	1.4354	1.6364	0.9699	0.060*	0.564 (6)
H23D	1.4092	1.6188	0.9286	0.060*	0.436 (6)
H23E	1.3584	1.6458	1.0241	0.060*	0.436 (6)
H23F	1.3202	1.7551	0.9337	0.060*	0.436 (6)
C24	1.1292 (9)	1.6561 (9)	0.8566 (6)	0.053 (3)	
H24A	1.0568	1.6040	0.8379	0.080*	0.564 (6)
H24B	1.1768	1.7262	0.8025	0.080*	0.564 (6)
H24C	1.0823	1.7047	0.9005	0.080*	0.564 (6)
H24D	1.0779	1.5941	0.8330	0.080*	0.436 (6)
H24E	1.2174	1.6865	0.8169	0.080*	0.436 (6)
H24F	1.0709	1.7375	0.8595	0.080*	0.436 (6)

### Atomic displacement parameters (Å^2^)

**Table:**

	*U*^11^	*U*^22^	*U*^33^	*U*^12^	*U*^13^	*U*^23^
Zn1	0.0175 (4)	0.0132 (4)	0.0153 (4)	−0.0021 (3)	−0.0029 (3)	−0.0061 (3)
S1	0.0279 (9)	0.0143 (8)	0.0187 (9)	−0.0024 (7)	−0.0078 (7)	−0.0056 (7)
S2	0.0245 (9)	0.0196 (9)	0.0275 (10)	−0.0028 (7)	−0.0038 (8)	−0.0111 (7)
S3	0.0274 (18)	0.0231 (18)	0.0235 (18)	−0.0044 (13)	0.0022 (14)	−0.0072 (14)
S3'	0.027 (2)	0.022 (2)	0.024 (2)	−0.0031 (16)	−0.0019 (17)	−0.0097 (17)
O1	0.019 (2)	0.009 (2)	0.028 (3)	−0.0014 (18)	−0.007 (2)	−0.0063 (19)
O2	0.023 (2)	0.015 (2)	0.027 (3)	−0.005 (2)	−0.006 (2)	−0.009 (2)
O3	0.030 (3)	0.025 (3)	0.027 (3)	−0.008 (2)	−0.004 (2)	−0.016 (2)
O4	0.110 (6)	0.026 (3)	0.072 (5)	−0.001 (4)	−0.061 (5)	−0.003 (3)
O1W	0.036 (3)	0.038 (3)	0.033 (3)	−0.009 (3)	−0.012 (2)	−0.008 (3)
N1	0.019 (3)	0.019 (3)	0.019 (3)	−0.003 (2)	−0.005 (2)	−0.008 (2)
N2	0.021 (3)	0.018 (3)	0.018 (3)	−0.002 (2)	−0.007 (2)	−0.012 (2)
N3	0.031 (3)	0.012 (3)	0.021 (3)	−0.002 (2)	−0.015 (3)	−0.007 (2)
N4	0.019 (2)	0.018 (2)	0.020 (3)	−0.004 (2)	−0.005 (2)	−0.013 (2)
N5	0.016 (3)	0.012 (3)	0.017 (3)	0.000 (2)	−0.009 (2)	−0.005 (2)
C1	0.015 (3)	0.018 (3)	0.020 (3)	−0.002 (3)	−0.006 (3)	−0.008 (3)
C2	0.011 (3)	0.016 (3)	0.019 (3)	−0.002 (2)	−0.002 (3)	−0.008 (3)
C3	0.018 (3)	0.017 (3)	0.019 (3)	−0.002 (3)	0.002 (3)	−0.009 (3)
C4	0.021 (3)	0.011 (3)	0.022 (4)	−0.007 (3)	0.005 (3)	−0.006 (3)
C5	0.017 (3)	0.015 (3)	0.019 (3)	−0.007 (3)	0.002 (3)	−0.004 (3)
C6	0.017 (3)	0.011 (3)	0.016 (3)	−0.002 (2)	0.003 (3)	−0.008 (3)
C7	0.016 (3)	0.015 (3)	0.012 (3)	−0.006 (3)	0.000 (3)	−0.001 (3)
C8	0.016 (3)	0.020 (3)	0.015 (3)	−0.005 (3)	0.001 (3)	−0.009 (3)
C9	0.018 (3)	0.015 (3)	0.023 (4)	0.004 (3)	−0.007 (3)	−0.009 (3)
C10	0.021 (3)	0.014 (3)	0.024 (4)	0.001 (3)	−0.007 (3)	−0.010 (3)
C11	0.022 (3)	0.013 (3)	0.021 (4)	−0.001 (3)	−0.004 (3)	−0.002 (3)
C12	0.019 (3)	0.015 (3)	0.019 (3)	−0.005 (2)	−0.007 (2)	−0.010 (2)
C14	0.024 (3)	0.015 (3)	0.020 (3)	−0.005 (3)	−0.002 (3)	−0.007 (3)
C15	0.016 (3)	0.020 (3)	0.025 (4)	−0.005 (3)	0.000 (3)	−0.014 (3)
C16	0.015 (3)	0.019 (3)	0.024 (4)	−0.006 (3)	−0.002 (3)	−0.012 (3)
C17	0.014 (3)	0.013 (3)	0.018 (3)	−0.007 (2)	−0.001 (3)	−0.009 (3)
C13	0.018 (3)	0.013 (3)	0.018 (3)	−0.003 (3)	−0.005 (3)	−0.011 (3)
C18	0.014 (3)	0.021 (3)	0.026 (4)	0.002 (3)	−0.008 (3)	−0.015 (3)
C19	0.013 (3)	0.016 (3)	0.023 (4)	0.003 (3)	−0.008 (3)	−0.009 (3)
C20	0.018 (3)	0.011 (3)	0.023 (3)	0.004 (3)	−0.005 (3)	−0.008 (3)
C21	0.029 (4)	0.020 (4)	0.035 (4)	−0.002 (3)	−0.004 (3)	−0.012 (3)
C22	0.038 (4)	0.026 (4)	0.026 (4)	−0.001 (3)	−0.009 (3)	−0.004 (3)
C23	0.043 (5)	0.037 (5)	0.042 (5)	−0.014 (4)	0.001 (4)	−0.014 (4)
C24	0.058 (6)	0.042 (5)	0.049 (6)	0.004 (5)	−0.026 (5)	0.012 (5)

### Geometric parameters (Å, °)

**Table:**

Zn1—O1	1.976 (4)	C6—C7	1.448 (9)
Zn1—N1	2.081 (5)	C7—H7	0.9500
Zn1—N5	2.143 (5)	C9—C10	1.381 (9)
Zn1—N4	2.157 (5)	C9—H9	0.9500
Zn1—S1	2.3542 (18)	C10—C11	1.373 (9)
S1—C8	1.744 (7)	C10—H10	0.9500
S2—O3	1.502 (5)	C11—C12	1.432 (7)
S2—C22	1.782 (7)	C11—H11	0.9500
S2—C21	1.790 (7)	C12—C14	1.3900
S3—O4	1.335 (7)	C12—C13	1.3900
S3—C23	1.825 (8)	C14—C15	1.3900
S3—C24	1.832 (8)	C14—H14	0.9500
S3—H23D	1.7249	C15—C16	1.3900
S3'—O4	1.342 (7)	C15—H15	0.9500
S3'—C23	1.777 (8)	C16—C17	1.3900
S3'—C24	1.834 (9)	C16—C18	1.427 (7)
O1—C1	1.329 (7)	C17—C13	1.3900
O2—C3	1.373 (8)	C18—C19	1.371 (9)
O2—H2	0.8400	C18—H18	0.9500
O1W—H1W1	0.8400	C19—C20	1.397 (9)
O1W—H1W2	0.8400	C19—H19	0.9500
N1—C7	1.282 (8)	C20—H20	0.9500
N1—N2	1.385 (7)	C21—H21A	0.9800
N2—C8	1.321 (8)	C21—H21B	0.9800
N3—C8	1.349 (8)	C21—H21C	0.9800
N3—H31	0.8800	C22—H22A	0.9800
N3—H32	0.8800	C22—H22B	0.9800
N4—C9	1.344 (8)	C22—H22C	0.9800
N4—C13	1.394 (6)	C23—H23A	0.9800
N5—C20	1.325 (8)	C23—H23B	0.9800
N5—C17	1.388 (6)	C23—H23C	0.9800
C1—C2	1.405 (8)	C23—H23D	0.9601
C1—C6	1.421 (9)	C23—H23E	0.9600
C2—C3	1.389 (9)	C23—H23F	0.9600
C2—H2A	0.9500	C24—H24A	0.9800
C3—C4	1.398 (9)	C24—H24B	0.9800
C4—C5	1.373 (9)	C24—H24C	0.9800
C4—H4	0.9500	C24—H24D	0.9599
C5—C6	1.413 (8)	C24—H24E	0.9600
C5—H5	0.9500	C24—H24F	0.9601
			
O1—Zn1—N1	89.26 (19)	C14—C12—C13	120.0
O1—Zn1—N5	104.25 (19)	C14—C12—C11	122.6 (4)
N1—Zn1—N5	94.6 (2)	C13—C12—C11	117.4 (4)
O1—Zn1—N4	95.47 (18)	C12—C14—C15	120.0
N1—Zn1—N4	172.4 (2)	C12—C14—H14	120.0
N5—Zn1—N4	78.52 (19)	C15—C14—H14	120.0
O1—Zn1—S1	143.98 (15)	C14—C15—C16	120.0
N1—Zn1—S1	82.37 (15)	C14—C15—H15	120.0
N5—Zn1—S1	111.28 (14)	C16—C15—H15	120.0
N4—Zn1—S1	97.11 (14)	C17—C16—C15	120.0
C8—S1—Zn1	93.9 (2)	C17—C16—C18	118.2 (4)
O3—S2—C22	105.7 (3)	C15—C16—C18	121.7 (4)
O3—S2—C21	105.6 (3)	N5—C17—C13	118.7 (3)
C22—S2—C21	97.8 (4)	N5—C17—C16	121.3 (3)
O4—S3—C23	111.0 (4)	C13—C17—C16	120.0
O4—S3—C24	107.3 (4)	C17—C13—C12	120.0
C23—S3—C24	93.9 (4)	C17—C13—N4	118.5 (3)
O4—S3—H23D	124.6	C12—C13—N4	121.5 (3)
C24—S3—H23D	111.8	C19—C18—C16	119.4 (6)
O4—S3'—C23	113.4 (5)	C19—C18—H18	120.3
O4—S3'—C24	106.8 (4)	C16—C18—H18	120.3
C23—S3'—C24	95.5 (4)	C18—C19—C20	119.2 (6)
C1—O1—Zn1	122.6 (4)	C18—C19—H19	120.4
C3—O2—H2	109.5	C20—C19—H19	120.4
S3—O4—S3'	57.4 (3)	N5—C20—C19	122.9 (6)
H1W1—O1W—H1W2	108.8	N5—C20—H20	118.5
C7—N1—N2	115.8 (5)	C19—C20—H20	118.5
C7—N1—Zn1	123.3 (4)	S2—C21—H21A	109.5
N2—N1—Zn1	119.9 (4)	S2—C21—H21B	109.5
C8—N2—N1	113.3 (5)	H21A—C21—H21B	109.5
C8—N3—H31	120.0	S2—C21—H21C	109.5
C8—N3—H32	120.0	H21A—C21—H21C	109.5
H31—N3—H32	120.0	H21B—C21—H21C	109.5
C9—N4—C13	119.3 (5)	S2—C22—H22A	109.5
C9—N4—Zn1	128.9 (4)	S2—C22—H22B	109.5
C13—N4—Zn1	111.7 (3)	H22A—C22—H22B	109.5
C20—N5—C17	119.0 (5)	S2—C22—H22C	109.5
C20—N5—Zn1	128.8 (4)	H22A—C22—H22C	109.5
C17—N5—Zn1	112.1 (3)	H22B—C22—H22C	109.5
O1—C1—C2	118.1 (6)	S3—C23—H23A	109.5
O1—C1—C6	124.0 (6)	S3'—C23—H23B	108.6
C2—C1—C6	117.8 (6)	S3—C23—H23B	109.5
C3—C2—C1	121.6 (6)	H23A—C23—H23B	109.5
C3—C2—H2A	119.2	S3'—C23—H23C	139.0
C1—C2—H2A	119.2	S3—C23—H23C	109.5
O2—C3—C2	117.8 (6)	H23A—C23—H23C	109.5
O2—C3—C4	121.4 (6)	H23B—C23—H23C	109.5
C2—C3—C4	120.8 (6)	S3'—C23—H23D	109.5
C5—C4—C3	118.0 (6)	S3'—C23—H23E	109.4
C5—C4—H4	121.0	S3—C23—H23E	132.4
C3—C4—H4	121.0	H23D—C23—H23E	109.5
C4—C5—C6	123.0 (6)	S3'—C23—H23F	109.6
C4—C5—H5	118.5	S3—C23—H23F	115.8
C6—C5—H5	118.5	H23D—C23—H23F	109.5
C5—C6—C1	118.5 (6)	H23E—C23—H23F	109.5
C5—C6—C7	116.5 (6)	S3—C24—H24A	109.5
C1—C6—C7	125.0 (6)	S3'—C24—H24A	110.7
N1—C7—C6	123.8 (6)	S3—C24—H24B	109.5
N1—C7—H7	118.1	S3'—C24—H24B	136.8
C6—C7—H7	118.1	H24A—C24—H24B	109.5
N2—C8—N3	116.1 (6)	S3—C24—H24C	109.5
N2—C8—S1	127.2 (5)	H24A—C24—H24C	109.5
N3—C8—S1	116.7 (5)	H24B—C24—H24C	109.5
N4—C9—C10	121.8 (6)	S3—C24—H24D	99.6
N4—C9—H9	119.1	S3'—C24—H24D	109.8
C10—C9—H9	119.1	S3—C24—H24E	76.3
C11—C10—C9	120.1 (6)	S3'—C24—H24E	109.4
C11—C10—H10	119.9	H24D—C24—H24E	109.5
C9—C10—H10	119.9	S3—C24—H24F	145.6
C10—C11—C12	119.8 (6)	S3'—C24—H24F	109.3
C10—C11—H11	120.1	H24D—C24—H24F	109.5
C12—C11—H11	120.1	H24E—C24—H24F	109.5
			
O1—Zn1—S1—C8	91.0 (3)	C1—C6—C7—N1	−11.7 (10)
N1—Zn1—S1—C8	12.9 (3)	N1—N2—C8—N3	−178.5 (5)
N5—Zn1—S1—C8	−79.0 (3)	N1—N2—C8—S1	2.1 (8)
N4—Zn1—S1—C8	−159.5 (3)	Zn1—S1—C8—N2	−13.0 (6)
N1—Zn1—O1—C1	−36.1 (5)	Zn1—S1—C8—N3	167.7 (5)
N5—Zn1—O1—C1	58.4 (5)	C13—N4—C9—C10	−0.1 (9)
N4—Zn1—O1—C1	137.9 (5)	Zn1—N4—C9—C10	176.3 (4)
S1—Zn1—O1—C1	−112.0 (5)	N4—C9—C10—C11	−1.0 (10)
C23—S3—O4—S3'	47.1 (4)	C9—C10—C11—C12	0.9 (9)
C24—S3—O4—S3'	−54.3 (4)	C10—C11—C12—C14	−179.9 (5)
C23—S3'—O4—S3	−49.9 (4)	C10—C11—C12—C13	0.2 (7)
C24—S3'—O4—S3	54.0 (4)	C13—C12—C14—C15	0.0
O1—Zn1—N1—C7	29.5 (5)	C11—C12—C14—C15	−179.9 (5)
N5—Zn1—N1—C7	−74.8 (5)	C12—C14—C15—C16	0.0
S1—Zn1—N1—C7	174.3 (5)	C14—C15—C16—C17	0.0
O1—Zn1—N1—N2	−161.9 (5)	C14—C15—C16—C18	177.5 (5)
N5—Zn1—N1—N2	93.9 (5)	C20—N5—C17—C13	176.9 (4)
S1—Zn1—N1—N2	−17.0 (4)	Zn1—N5—C17—C13	−7.0 (4)
C7—N1—N2—C8	−177.1 (6)	C20—N5—C17—C16	−1.7 (6)
Zn1—N1—N2—C8	13.4 (7)	Zn1—N5—C17—C16	174.5 (2)
O1—Zn1—N4—C9	74.9 (5)	C15—C16—C17—N5	178.5 (4)
N5—Zn1—N4—C9	178.4 (5)	C18—C16—C17—N5	0.9 (5)
S1—Zn1—N4—C9	−71.3 (5)	C15—C16—C17—C13	0.0
O1—Zn1—N4—C13	−108.5 (4)	C18—C16—C17—C13	−177.6 (4)
N5—Zn1—N4—C13	−5.0 (3)	N5—C17—C13—C12	−178.5 (4)
S1—Zn1—N4—C13	105.3 (3)	C16—C17—C13—C12	0.0
O1—Zn1—N5—C20	−85.1 (5)	N5—C17—C13—N4	2.6 (5)
N1—Zn1—N5—C20	5.2 (5)	C16—C17—C13—N4	−178.8 (4)
N4—Zn1—N5—C20	−177.9 (6)	C14—C12—C13—C17	0.0
S1—Zn1—N5—C20	88.8 (5)	C11—C12—C13—C17	179.9 (4)
N1—Zn1—N5—C17	−170.5 (3)	C14—C12—C13—N4	178.8 (4)
N4—Zn1—N5—C17	6.3 (3)	C11—C12—C13—N4	−1.3 (5)
S1—Zn1—N5—C17	−86.9 (3)	C9—N4—C13—C17	−180.0 (4)
Zn1—O1—C1—C2	−154.9 (4)	Zn1—N4—C13—C17	3.1 (4)
Zn1—O1—C1—C6	26.7 (8)	C9—N4—C13—C12	1.2 (7)
O1—C1—C2—C3	177.1 (6)	Zn1—N4—C13—C12	−175.7 (2)
C6—C1—C2—C3	−4.4 (9)	C17—C16—C18—C19	0.4 (7)
C1—C2—C3—O2	−179.0 (6)	C15—C16—C18—C19	−177.2 (4)
C1—C2—C3—C4	0.2 (10)	C16—C18—C19—C20	−0.9 (9)
O2—C3—C4—C5	−178.2 (6)	C17—N5—C20—C19	1.1 (9)
C2—C3—C4—C5	2.6 (9)	Zn1—N5—C20—C19	−174.3 (5)
C3—C4—C5—C6	−1.1 (10)	C18—C19—C20—N5	0.2 (10)
C4—C5—C6—C1	−3.1 (9)	O4—S3'—C23—S3	45.0 (4)
C4—C5—C6—C7	176.9 (6)	C24—S3'—C23—S3	−66.0 (3)
O1—C1—C6—C5	−175.9 (6)	O4—S3—C23—S3'	−44.3 (4)
C2—C1—C6—C5	5.7 (9)	C24—S3—C23—S3'	65.9 (3)
O1—C1—C6—C7	4.1 (10)	O4—S3—C24—S3'	49.6 (4)
C2—C1—C6—C7	−174.4 (6)	C23—S3—C24—S3'	−63.8 (3)
N2—N1—C7—C6	179.2 (5)	O4—S3'—C24—S3	−49.1 (4)
Zn1—N1—C7—C6	−11.8 (9)	C23—S3'—C24—S3	67.4 (3)
C5—C6—C7—N1	168.3 (6)		

### Hydrogen-bond geometry (Å, °)

**Table:**

*D*—H···*A*	*D*—H	H···*A*	*D*···*A*	*D*—H···*A*
O2—H2···O3	0.84	1.84	2.679 (6)	172
N3—H31···N2^i^	0.88	2.11	2.984 (8)	174
N3—H32···O4	0.88	2.33	3.074 (8)	143
O1w—H1w1···O1^ii^	0.84	2.11	2.911 (7)	160
O1w—H1w2···O2	0.84	2.25	3.055 (7)	161

Symmetry codes: (i) −*x*+2, −*y*+2, −*z*+2; (ii) −*x*+1, −*y*+2, −*z*+1.
